# Conductance tomography of conductive filaments in intrinsic silicon-rich silica RRAM†
†Electronic supplementary information (ESI) available. See DOI: 10.1039/c5nr04982b



**DOI:** 10.1039/c5nr04982b

**Published:** 2015-10-20

**Authors:** Mark Buckwell, Luca Montesi, Stephen Hudziak, Adnan Mehonic, Anthony J. Kenyon

**Affiliations:** a Department of Electronic and Electrical Engineering , University College London , Torrington Place , London WC1E 7JE , UK . Email: m.buckwell@ee.ucl.ac.uk ; Email: t.kenyon@ucl.ac.uk

## Abstract

We present results from an imaging study of filamentary conduction in silicon suboxide resistive RAM devices. We used a conductive atomic force microscope to etch through devices while measuring current, allowing us to produce tomograms of conductive filaments. To our knowledge this is the first report of such measurements in an intrinsic resistance switching material.

## Abstract 2

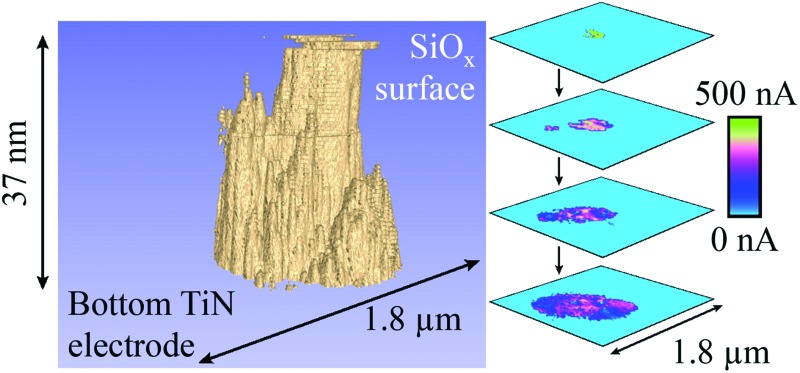
Conductive atomic force microscopy was used to etch through SiO_*x*_ resistance switching devices to produce three-dimensional renderings of conductive filaments.

Resistance switching memories, or resistive RAM (RRAM) devices, are a next-generation solution to the problems of scaling existing semiconductor memories such as flash to smaller dimensions.^1^ RRAM devices are simple, two terminal devices that can be switched between non-volatile conductance states with a resistance contrast of several orders of magnitude.^2^ While the formation of conductive filaments by metal diffusion from electrochemically active electrodes (so-called electrochemical metallisation or conductive bridge RAM (CBRAM)) is one widely studied approach, such systems present difficulties for integration into CMOS technologies due to the possibility of detrimental metal ion diffusion.^3^ Materials in which resistance switching is intrinsic, conversely, do not pose such risks and may be more suitable for application in commercial, consumer devices.

A particularly interesting class of devices are those consisting of an amorphous, insulating oxide sandwiched between inert electrodes. The mechanisms proposed to explain intrinsic resistance switching in such devices rely on redox reactions and oxygen migration,^4^ and this class of device is often referred to as valence change memory (VCM).^2^ The field-driven formation of a conductive chain of oxygen vacancies bridges the insulating layer and switches the initially insulating oxide into a more conductive state.

The switching mechanism in CBRAM is relatively well understood and such devices have displayed promising characteristics such as large on/off current ratios, high endurance and low device variability.^4^ Conversely, the switching mechanism in VCM devices is more ambiguous and must be better understood if devices are to be optimised in order to deliver competitive characteristics.

Additional insight into the nature of the conductive filament in VCM devices would certainly aid optimisation. However, the intrinsic filamentation process has only been studied indirectly. Detailed information on the size and shape of filaments has been obtained from imaging studies of extrinsic (CBRAM) systems^5,6^ along with devices exhibiting surface-confined switching.^7^ In intrinsic systems such measurements are more difficult, principally due to the poor electron microscopy contrast between conductive oxygen vacancies and the surrounding oxide.

There are, to our knowledge, no reports of three-dimensional filament visualisation in intrinsic oxide RRAM. In this letter we present recent work in which we have been able to produce, for the first time, tomographic images of intrinsic conductive filaments in oxide RRAM devices. Our approach is similar that of Celano *et al.* ^5^ in which conductive atomic force microscopy was used to etch through oxide layers while simultaneously mapping conductivity. Crucially, while Celano's study was on an extrinsic conductive bridge system in which copper ions were diffused through a layer of aluminium oxide, our devices exhibit intrinsic resistance switching behaviour, without the need for metallic dopants.^3^ We are also able to confirm for the first time multiple filament growths in an intrinsic VCM material, with filaments ‘competing’ to bridge the dielectric layer.

We have proposed previously that filamentation may be partly reliant on the presence of intrinsic structural defects such as growth column boundaries within the dielectric that could facilitate the migration of oxygen.^3^ Such inhomogeneities have been observed in amorphous oxides in the form of vertical columnar structures.^8^ Thus there may be particular locations within dielectric films that are predisposed to filamentation under electrical stress. Here we show that this inhomogeneous oxide structure is reflected in the shape and internal structure of the conductive filaments.

Our devices were fabricated on p-type silicon substrates with a 4 μm thermally grown oxide. 100 nm thick titanium nitride bottom and top electrodes were sputtered either side of a 37 nm thick SiO_*x*_ active layer. All layers were deposited in an argon atmosphere, with the SiO_*x*_ co-sputtered from silicon and SiO_2_ targets. This produced a sub-stoichiometric, silicon-rich film with *x* ≈ 1.3 (confirmed by X-ray photoelectron spectroscopy). The top electrodes were subsequently patterned into individual square devices with side lengths ranging from 10 to 400 μm. Following fabrication, devices were annealed at 600 °C in an inert atmosphere. Our device configuration is shown schematically in Fig. 1(a).

**Fig. 1:**
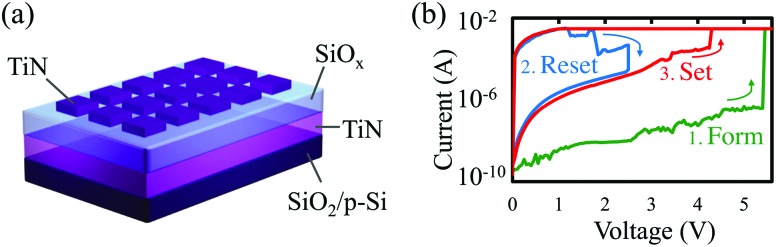
The structure and electrical characteristics of our devices. (a) Device schematic showing our individually patterned TiN/SiO_*x*_/TiN devices on a SiO_2_/p-Si substrate. (b) Typical switching characteristics for our metal–insulator–metal devices. Following an electroforming sweep (1), the device may be reset (2) and set (3) repeatedly to cycle between ‘on’ and ‘off’ states. The state of the device may be read by applying a small bias, usually 0.5 V. At this voltage the resistance contrast between the ‘on’ and ‘off’ states is around three orders of magnitude. A larger version of this figure is shown in the ESI, Fig. S1.†

We carried out electrical measurements under ambient conditions using a Keithley 4200-SCS semiconductor characterisation system and tungsten needle probes with a nominal footprint diameter of 1 μm. The presence of deposited electrodes restricts the probing of the sandwiched layer, so once we had characterised the resistance switching of our devices we used a tungsten needle as a top electrode on the SiO_*x*_ surface away from TiN top contacts to enable us to directly study the oxide below the contact point.

We performed conductive atomic force microscopy (CAFM) with a Bruker Icon Microscope. CAFM scans were carried out using boron-doped-diamond coated silicon cantilevers with a nominal spring constant of 6 N m^–1^. CAFM data were processed using NanoScope Analysis v1.5 and three-dimensional rendering was done using 3D Slicer v4.4.0.^9,10^

Fig. 1(b) shows typical switching characteristics of a TiN/SiO_*x*_/TiN stack. Beginning in a highly insulating pristine state, the device is electroformed at around 6 V into the ‘on’ state. This corresponds to the formation of a conductive filament. Following this, the state may be switched ‘off’ into an intermediate state and subsequently cycled between on and off at around 3 V and 2.2 V, respectively. A current compliance level of 3 mA is set during electroforming and switching to the on state in order to limit the energy input to the system. Without this limit, RRAM devices tend to break down irreversibly as current-induced Joule heating at high fields induces destructive melting and mixing of layers.^10^ To switch the device into the off state the compliance is removed, allowing Joule heating at a low field to rupture the filament through reoxidation of some of the oxygen vacancies as the local oxygen mobility increases.^12^ The device never returns to its initial, pristine resistance state as the filament is only partially reoxidised.^11^ This switching behaviour is known as unipolar, as opposed to bipolar switching in which opposite biases are used to switch the device on and off.^1^ In both classes of device, the on/off state is read using a low voltage, generally below 1 V, to ascertain whether the resistance is low or high.

We have previously shown both types of behaviour in older device generations with asymmetric p-Si and ITO electrodes.^3,11^ However, in symmetric metal–insulator–metal devices we found that only unipolar operation was successful,^13^ therefore we concentrated on this for the work described here. Unipolar switching is also often termed a thermochemical mechanism (TCM).^4^

Here, we have studied filaments in the on state following a single electroforming operation with either a positive or negative bias. We did this in order to focus on the changes occurring in the dielectric during the initial forming state of device operation. It should be mentioned that filamentation in both the on and off state have been observed in CBRAM,^5,6^ surface switching^7^ and bipolar VCM^14^ devices but not in unipolar VCM devices.

To probe filamentation in our SiO_*x*_ we first generated conductive filaments by applying electroforming voltages with a tungsten needle placed on the surface of the film. We have previously found that, due to native oxidation of the pristine SiO_*x*_ surface, higher fields are required than for electroforming with a top electrode.^7^ Thus we set current compliance below 3 mA in order to limit thermal effects and prevent hard breakdown of the oxide under voltage stresses of up to ±20 V. As an additional means of reducing the duration of the electrical stress, voltages were applied as fast pulses, in effect very fast sweeps. The resistance state of the filament was read with 0.6 V after the pulse to ensure electroforming had occurred. Typically, compliance currents greater than 1 mA produced surface distortions that were clearly visible under an optical microscope. Such features are undesirable as they indicate damage that would not normally occur during regular operation at lower fields with a 3 mA current compliance.

We then used CAFM to locate individual filaments by detecting points of high current in otherwise-insulating regions. We noted the position of the tungsten probe in relation to the patterned top electrodes from full TiN/SiO_*x*_/TiN device stacks and then scanned 30 μm by 30 μm square regions to locate the conductive spot corresponding to the filament. For these scans the bottom electrode of the device was biased at 2 V relative to the CAFM tip, which was held at 0 V. Such a high scanning bias was needed to overcome the tunnelling gap present between the tip and sample when scanning with a low setpoint.

We have previously observed that electroforming locations show structural deformation of the SiO_*x*_ surface.^13^ Such structures are elastic and have been attributed to outgassing of oxygen and thermal expansion of the surface during electroforming.^13,15–17^ Note that the structure and conductivity of such spots persists for at least 48 hours after electroforming. In this work we looked only at smaller, few nm distortions, a typical example of which is shown in the ESI, Fig. S2.†

Once a filament had been located, the setpoint voltage of the CAFM cantilever was raised in order to press the tip into the sample. To ensure sufficient pressure for the removal of material with high lateral resolution in the current map, the setpoint voltage was lowered to just below the point at which detail in the current map was washed out by the contact pressure. Note that the setpoint voltage needed for this condition varied between sampling locations and CAFM tips, most likely due to differences in tip geometry. For all scans the tip velocity was kept below 10 μm s^–1^.

For depth profiling scans the bottom electrode bias was reduced to 50 mV relative to the CAFM tip because the tip was being forced into close contact with the sample. A series resistance of 178 kΩ was used at all times to reduce transient charging and discharging of surface defect states. The maximum measured current in the filaments was around 500 nA with this setup. By continually raster scanning over the same area with a constant setpoint we were able to collect a data set of consecutive slices mapping current in two dimensions as material was scraped away from the SiO_*x*_ surface. Each image slice had a resolution of 256 × 256 pixels, giving a lateral resolution of 3.1 to 7.0 nm, depending on the image size. This process is shown schematically in Fig. 2. The number of slices obtained for each filament varied greatly, most likely as a result of variations in tip geometry, scan size and tip velocity.

**Fig. 2:**
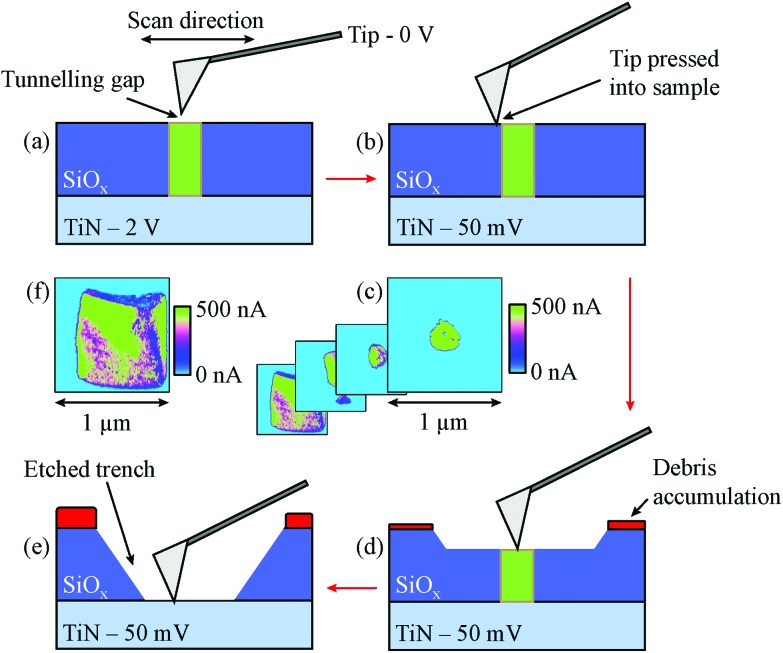
Schematic representation of the depth profiling process. (a) Filaments are located by performing a CAFM scan with a low setpoint voltage to detect conductive spots in insulating areas. Due to the tunnelling gap in these scans, a relatively high scanning bias of 2 V is applied between the tip and sample. (b) Once the filament has been located, the setpoint voltage is increased, causing the feedback system of the microscope to push the cantilever tip into the sample surface. The applied bias is also lowered to 50 mV as the tunnelling gap is removed. (c) and (d) As the region is raster scanned, a two-dimensional current map is acquired. Through repeated scanning of the same area, a trench is etched as material is removed from the SiO_*x*_ and accumulated at the edge of the scan region. This results in a set of current map image slices through the active layer that may be later rendered into a tomographic image. (e) Eventually the CAFM tip reaches the bottom TiN electrode. (f) At the bottom of the final trench the current map becomes roughly a square, corresponding to a scan of the conductive electrode.

Following data acquisition, current map slices were stacked vertically to produce a three-dimensional volume map of current in the SiO_*x*_. An anisotropic diffusion filter was applied to the volume data to reduce noise and smooth the transition between slices while preserving lateral data features in each slice. The volume was then rendered and the filament exposed within the bulk insulating material by thresholding the data to remove the background current noise of around 75 pA. This process left a solid three-dimensional rendering of the filament. For clarity and completeness, ESI Fig. S7† demonstrates this process for a pristine SiO_*x*_ sample, showing that there are no conductive filaments present, and Fig. S8† shows the effect of different threshold levels to a rendering. Note that in all figures showing a filament the corresponding current map slices have not had any thresholding applied. This has been done in order to clearly show the raw conductivity data.

Fig. 3(a) displays a tomographic rendering of the conductivity slices for a filament formed with +20 V at a current compliance of 0.1 μA. Interestingly, contrary to some suggestions in the literature,^18,19^ this filament does not have a conical shape but is instead tubular. It is also evident that there are lateral variations in the current within the filament, possibly a reflection of the intrinsic columnar structure of sputtered amorphous oxides.^11^ Fig. 3(b)–(e) show conductive AFM cross-sections depicting the internal structure of the filament. These reveal that the current varies across the filament, with the most conductive regions located more centrally, and there are some regions in which no current flows. This suggests that the filament has an internal structure that follows many tightly packed paths through the SiO_*x*_. Therefore, rather than a single filament it is possible that we are instead observing a highly localised group of filaments that have fused together.

**Fig. 3:**
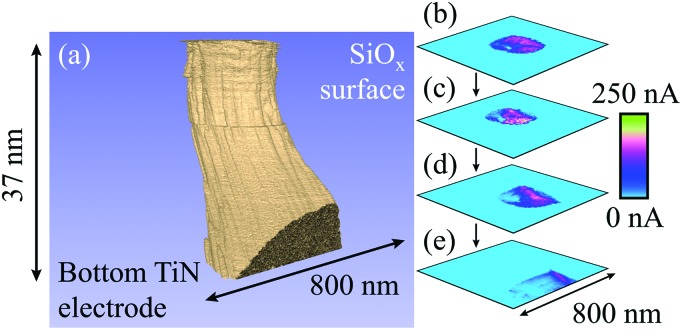
Representation of a filament formed with +20 V at a current compliance of 0.1 μA. (a) Tomographic rendering of the filament, in which conductive regions are shown in beige and the surrounding, insulating oxide is shown in blue. It has a remarkably tubular shape that reflects an underlying, smaller columnar structure. (b) to (e) CAFM cross-sections at equal intervals through the switching layer showing the internal structure of the filament, with clear lateral variations in current at different depths into the SiO_*x*_. Note that there is some drift evident in the cross-sections that is likely instrumental and caused by the large forces applied to the sample during scanning. As a result of the drift the filament intercepts the edge of the scan region, causing a flattening of its lower edge. The rendering in (a) has been shown from an angle that best displays the columnar structure rather than the drift. 474 slices with a lateral resolution of 3.1 nm were used in this figure. A larger version of this figure is shown in the ESI, Fig. S3.†

Fig. 4 presents conductive AFM measurements of a filament electroformed with –20 V at a current compliance of 0.1 μA. As the rendering in Fig. 4(a) shows, in this case the filament shape is more similar to the conical models in the literature. Again, there is an underlying columnar structure. It also appears that the conical shape is composed of multiple outer conductive pathways surrounding the main body of the filament. As noted for the positive filament in Fig. 3, it is possible that the structure is a collection of closely packed, smaller filaments that are bunched around a preferential pathway through the oxide. As shown in Fig. 4(b)–(e), the filament has a complex internal structure in which some regions are more conductive than others. Interestingly, although Fig. 4(a) and (b) show that the central region is the most conductive near the top of the filament, Fig. 4(d) and (e) demonstrate that it is actually the outer region that shows the greatest current towards the filament base.

**Fig. 4:**
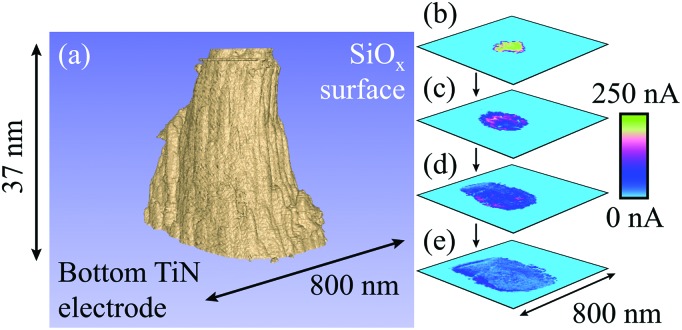
Representation of a filament formed with –20 V at a current compliance of 0.1 μA. (a) Tomographic rendering of the filament, in which conductive regions are shown in beige and the surrounding, insulating oxide is shown in blue. The shape is more conical than for the positively formed filament, yet there is still an underlying structure that reflects the intrinsic graininess of the SiO_*x*_. (b) to (e) CAFM cross-sections showing the internal variations in the filament current at different depths into the SiO_*x*_. 224 slices with a lateral resolution of 3.1 nm were used in this figure. A larger version of this figure is shown in the ESI, Fig. S4.†

Fig. 5 shows our second observation of a filament formed with a positive bias. In this case we have used a voltage of 12.5 V and current compliance of 1 mA. The filament shows a more conical structure than that of Fig. 3. There also appears to be a small second peak adjoining the main filament, with the pair of growths originating from a larger, roughly square region. However, we believe this second peak is most likely the appearance of the bottom electrode during etching, angled away from the horizontal due either to an uneven deposition or a scanning artefact. Notably, we are again able to observe a correlation between conductivity and the granular structure of the SiO_*x*_ in the filament. Fig. 5(b)–(e) show cross-sections through the filaments, highlighting the gradual appearance of the electrode and its eventual washout of the entire scan area. As already observed, the most conductive regions of the filament are located centrally. Interestingly, however, there appears to be a distinct split through the centre of the conductive region. This is particularly evident in Fig. 4(d), and is likely to be related to the appearance of the bottom electrode, which should be more conductive than the filament.

**Fig. 5:**
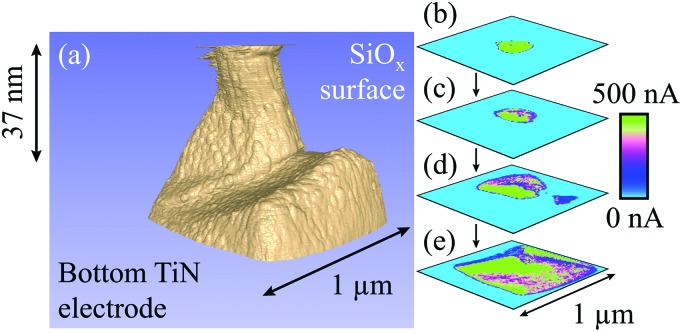
Representation of a filament formed with +12.5 V at a current compliance of 1 mA. (a) Tomographic rendering of the filament, in which conductive regions are shown in beige and the surrounding, insulating oxide is shown in blue. Near to the bottom electrode there is a branching of the growth trajectory, resulting in a pair of filament peaks. Only one of these bridges the entire active layer, however and the second is likely to be the appearance of the bottom TiN electrode. (b) to (e) Selected cross-sections through the SiO_*x*_, showing the emergence of the bottom electrode and its convergence with the primary growth pathway. Again, the internal, inhomogeneous structure of the filament is evident. 513 slices with a lateral resolution of 3.9 nm were used in this figure. A larger version of this figure is shown in the ESI, Fig. S5.†

It has been suggested in the literature that the filamentation process may not be only the direct generation of a single conductive pathway. Instead, structural inhomogeneities in the switching layer may present many alterative preferential routes for growth.^18,20,21^ Thus filaments may ‘compete’ to bridge the switching layer, with only a single filament in the end shorting the electrodes. This behaviour has previously been confirmed in CBRAM devices^22,23^ and we are now able to present findings that support the suggestion of multiple filamentation in intrinsic VCM systems.

Fig. 4(a) shows a spreading of conductive pathways from the main filament and may be evidence of multiple filaments. However, Fig. 6 demonstrates a much clearer observation we have made of this phenomenon. A voltage of –16 V at a current compliance of 1 μA was used here to electroform the oxide. It is clear that, aside from the main filament that reaches across the SiO_*x*_ there are several additional growths reaching through different portions of the oxide. Fig. 6(b)–(e) highlight the gradual appearance of multiple filaments through the film, and their convergence as the CAFM tip approaches the bottom electrode. The internal structure of the conductive pathway is evident here, with more conductive regions appearing more centrally in the filament.

**Fig. 6:**
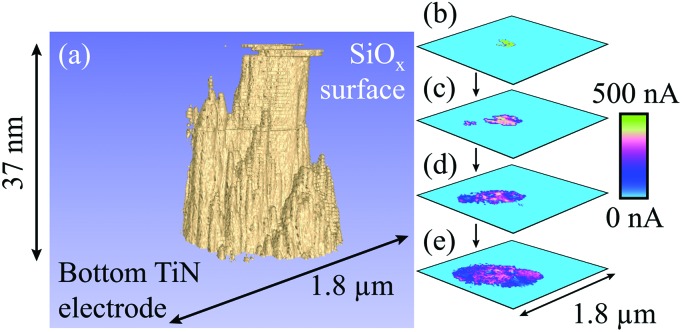
Representation of a filament formed with –16 V at a current compliance of 1 μA. (a) Tomographic rendering of a multiple filament, in which conductive regions are shown in beige and the surrounding, insulating oxide is shown in blue. There appear to be at least three constituents to the overall growth, although the smallest of these is only a few nanometres high. The main filament is very straight, although it becomes slightly conical as it approaches the bottom electrode. Note that a single slice has been removed due to high levels of noise that saturated the scan area. (b) to (e) Selected cross-sections through the filament, showing the appearance and convergence of multiple filaments. 116 slices with a lateral resolution of 7.0 nm were used in this figure. A larger version of this figure is shown in the ESI, Fig. S6.†

The presence of multiple filaments, as shown in Fig. 6, suggests that many partial filaments may be produced during electroforming. We have only observed those that occur local to the main filament, yet it is likely that other, more distant locations in the film between the electrodes will experience small growths during electroforming. This may be particularly true of device with larger top electrode areas than the nominally 1 μm footprint of our tungsten probes. It is reasonable to speculate that only a single growth ‘wins’ the race across the oxide, as the electric field will collapse once the electrodes are shorted. At this point there should be no further driving force to grow any secondary filaments. However, with repeated device cycling, the electrical stresses used may further develop competing filaments. It is also possible that some growth paths may not be suitable for filamentation, allowing conductive regions to grow some way into the active layer but not to fully bridge it.

It has previously been suggested that filament size is dependent upon the magnitude and duration of the applied bias.^20^ All the filaments we have so far observed are relatively large compared to the predictions in the literature of filament sizes of nanometres to tens of nanometres.^5,11,24,25^ Furthermore, successful resistance switching behaviour has been observed in other materials in smaller lateral areas than the cross-sectional areas of our filaments.^24–27^ It is also of note that we too have observed conductive regions at the SiO_*x*_ surface with sizes of the order of tens of nanometres.^8^ It therefore seems possible that filaments may continue to grow laterally as multiple conduction paths converge, until the applied bias is removed. This is likely to have been the case in our filaments as a result of the relatively high fields employed and the time taken for current compliance to reduce the applied voltage, even for a short pulse. A further consequence of this may be that the observed shape of the filaments is not representative of their shape at the time of forming. Additional growth before the voltage is removed may explain the deviation of the filaments from their expected shape. Notably, the power dissipation in unipolar devices is relatively high, which may explain the much smaller filaments observed using transmission electron microscopy in bipolar systems.^14^

An interesting aspect of the filaments we have observed is that their lateral sizes all appear similar, in the order of hundreds of nanometres, despite the application of different electroforming voltages and current compliances. We have previously seen that the conductance of the filament increases in accordance with the maximum applied voltage during a voltage sweep and have attributed this to a thickening of the filament.^28^ Although a more detailed investigation is required, it is puzzling that there is not an apparent correlation between the electroforming conditions and the lateral size of the filament.

It is important to note that C-AFM measurements undertaken as described in this work do not measure only conductivity; rather, they measure the connectedness of the exposed sample surface to the bottom electrode. If there is a very highly conductive layer, or set of filaments, near the surface but not connected (unless by tunnelling) to the bottom electrode, these will not be visible using our technique. There is therefore an inbuilt asymmetry in our measurements.

## Conclusions

The results we have presented here confirm the filamentary nature of conductive pathways in an intrinsic oxide RRAM device. We have demonstrated for the first time that the formation process follows the intrinsic columnar structure of the amorphous material. We have also been able to demonstrate for the first time that filament formation in VCM systems does not always follow a single pathway and that several filaments may grow simultaneously in a device.
