# Supplementary material for: Conductance tomography of conductive filaments in intrinsic silicon-rich silica RRAM
Source: Nanoscale. 2015 Oct 20;7(43):18030–5. doi: 10.1039/c5nr04982b (PMC4718172; doi:10.1039/c5nr04982b)
Supplement: Supplementary file 1 [file NR-007-C5NR04982B-s001.pdf]

Electronic Supplementary Material (ESI) for Nanoscale.
This journal is © The Royal Society of Chemistry 2015

Nanoscale

COMMUNICATION

Supplementary Information

Conductance tomography of conductive filaments in intrinsic silicon-rich silica RRAM

Mark Buckwell[†], Luca Montesi, Stephen Hudziak, Adnan Mehonic, and Anthony J. Kenyon[†]

Department of Electronic and Electrical Engineering, University College London, Torrington Place, London WC1E 7JE, United Kingdom
† E-mail: m.buckwell@ee.ucl.ac.uk, t.kenyon@ucl.ac.uk

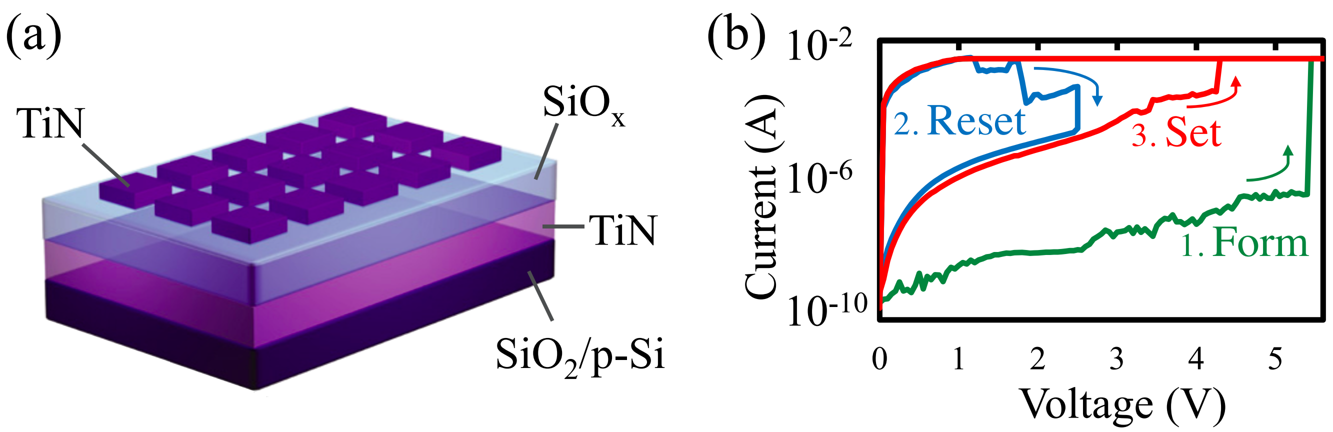

Fig. S1. Enlarged version of Fig. 1, the structure and electrical characteristics of our devices. (a) Device schematic showing our individually patterned TiN/SiOx/TiN devices on a SiO2/p-Si substrate. (b) Typical switching characteristics for our metal-insulator-metal devices. Following an electroforming sweep (1), the device may be reset (2) and set (3) repeatedly to cycle between 'on' and 'off' states. The state of the device may be read by applying a small bias, usually 0.5 V. At this voltage the resistance contrast between the 'on' and 'off' states is around three orders of magnitude.

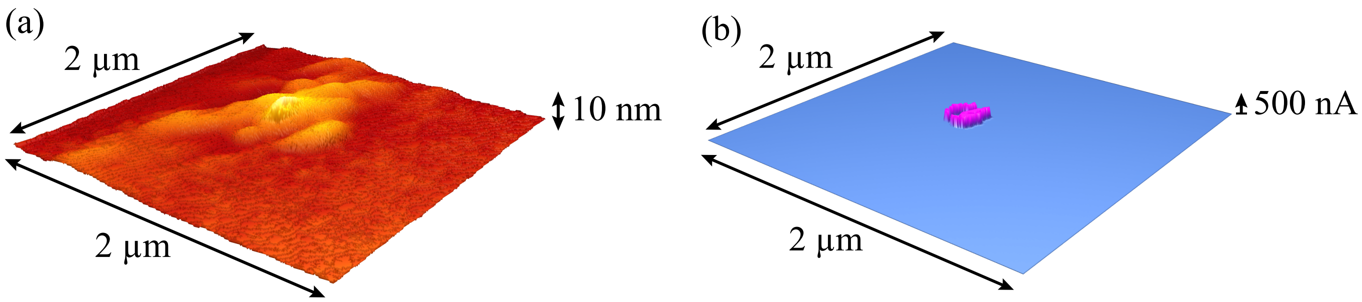

Fig. S2. Typical surface distortions observed as a result of landing a tungsten needle on pristine SiO_x and applying a voltage bias. (a) AFM tomography of the biased region, showing a small surface distortion. (b) Correlation of CAFM conductivity with the surface distortion in (a). This feature corresponds to the filament shown in Figs. 5 and S5.

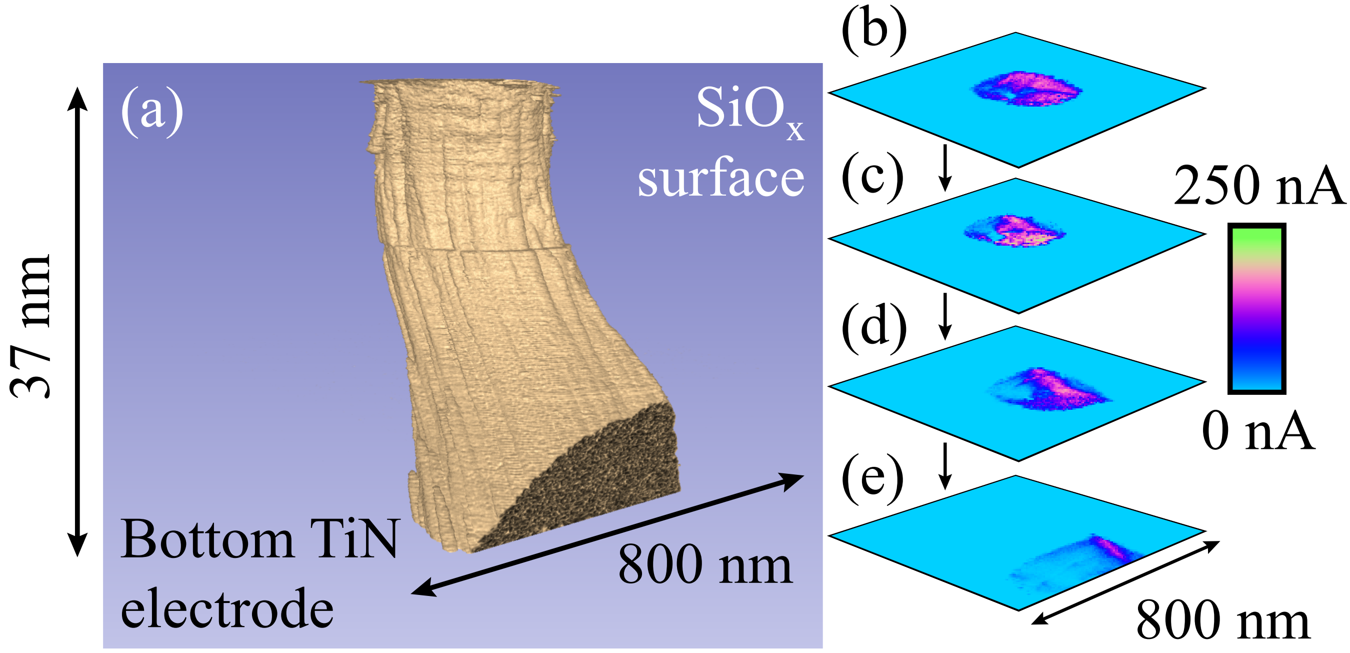

Fig. S3. Enlarged version of Fig. 3, representation of a filament formed with +20 V at a current compliance of 0.1 µA. (a) Tomographic rendering of the filament, in which conductive regions are shown in beige and the surrounding, insulating oxide is shown in blue. It has a remarkably tubular shape that reflects an underlying, smaller columnar structure. (b) to (e) CAFM cross-sections at equal intervals through the switching layer showing the internal structure of the filament, with clear lateral variations in current at different depths into the SiOx. Note that there is some drift evident in the cross-sections that is likely instrumental and a result of the large forces applied to the sample during scanning. As a result of the drift the filament intercepts the edge of the scan region, causing a flattening of its lower edge. The rendering in (a) has been shown from an angle that best displays the columnar structure rather than the drift. 474 slices with a lateral resolution of 3.1 nm were used in this figure.

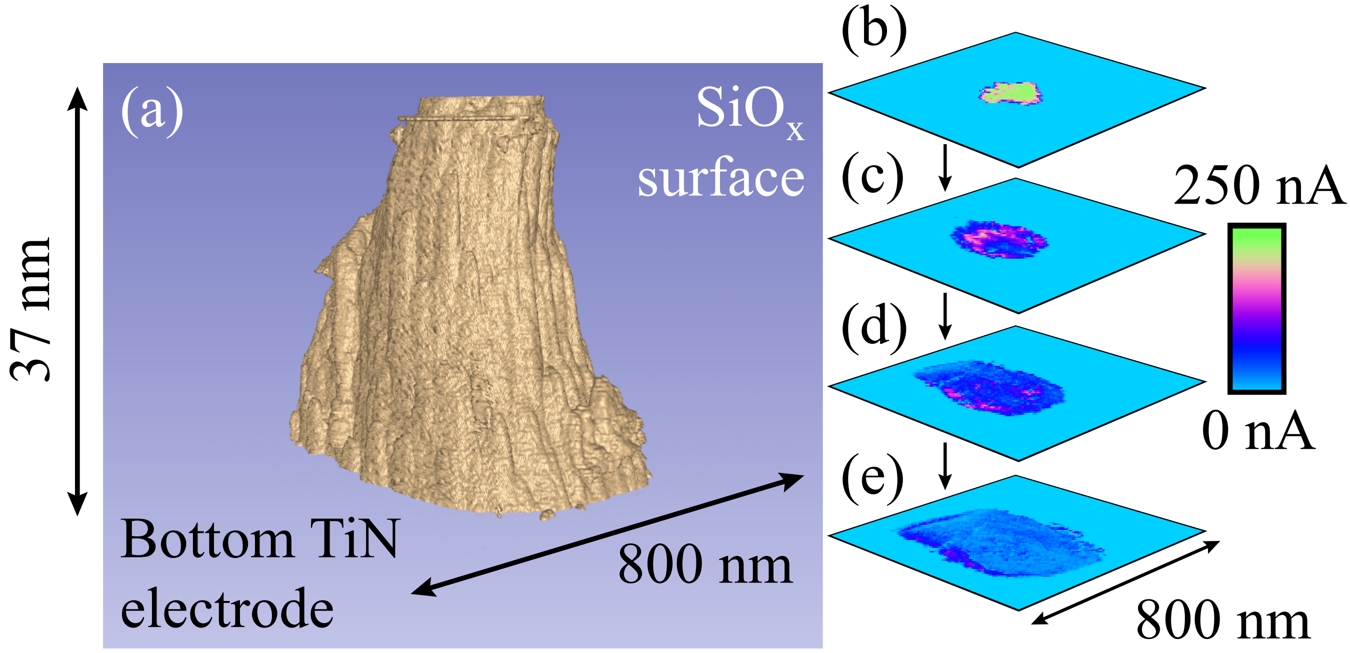

Fig. S4. Enlarged version of Fig. 4, representation of a filament formed with -20 V at a current compliance of 0.1 µA. (a) Tomographic rendering of the filament, in which conductive regions are shown in beige and the surrounding, insulating oxide is shown in blue. The shape is more conical than for the positively formed filament, yet there is still an underlying structure that reflects the intrinsic graininess of the SiO_x. (b) to (e) CAFM cross-sections showing the internal variations in the filament current at different depths into the SiOx. 224 slices with a lateral resolution of 3.1 nm were used in this figure.

 This journal is © The Royal Society of Chemistry 2015

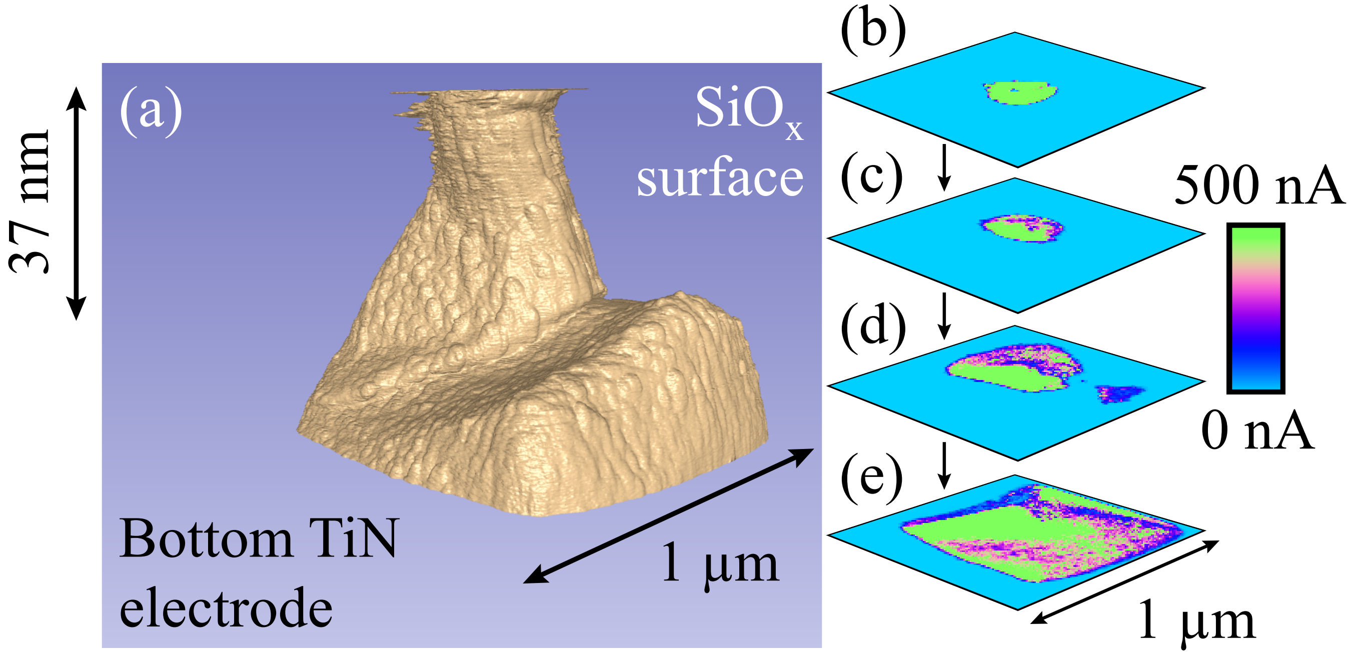

Fig. S5. Enlarged version of Fig. 5, representation of a filament formed with +12.5 V at a current compliance of 1 mA. (a) Tomographic rendering of the filament, in which conductive regions are shown in beige and the surrounding, insulating oxide is shown in blue. Near to the bottom electrode there is a branching of the growth trajectory, resulting in a pair of filament peaks. Only one of these bridges the entire active layer, however and the second is likely to be the appearance of the bottom TiN electrode. (b) to (e) Selected cross-sections through the SiO_x, showing the emergence of the bottom electrode and its convergence with the primary growth pathway. Again, the internal, inhomogeneous structure of the filament is evident. 513 slices with a lateral resolution of 3.9 nm were used in this figure.

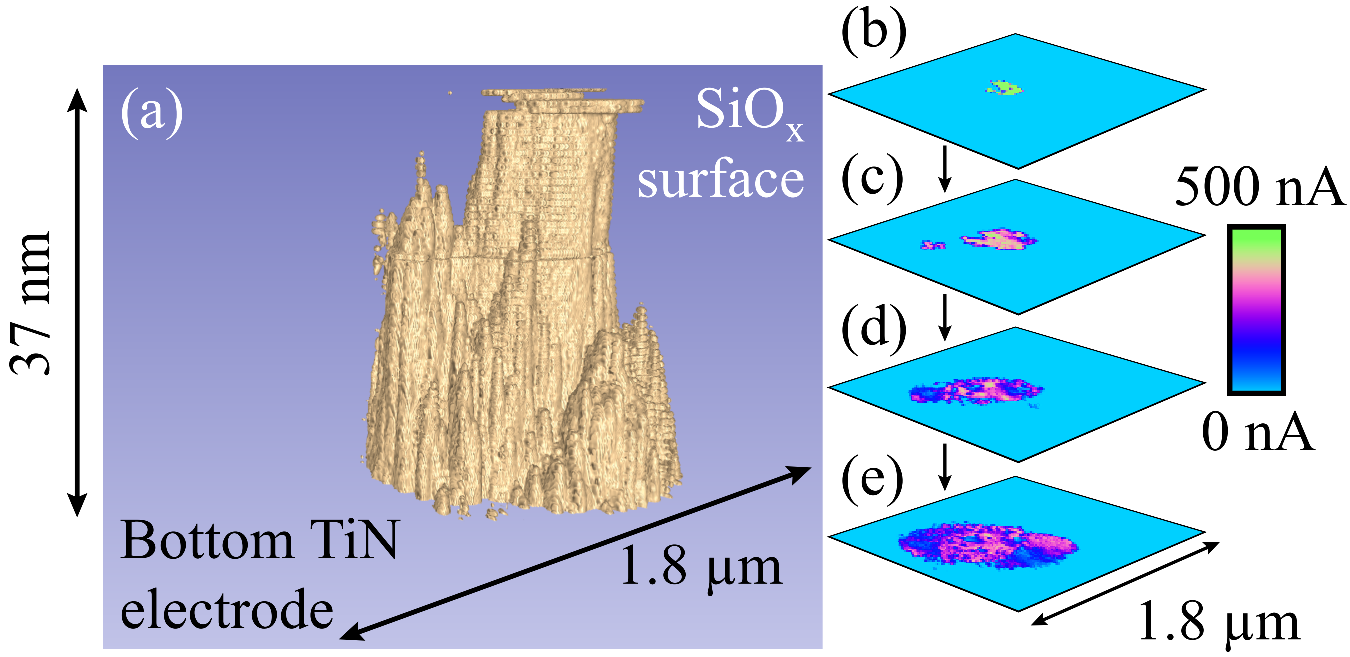

Fig. S6. Enlarged version of Fig. 6, representation of a filament formed with -16 V at a current compliance of 1 µA. (a) Tomographic rendering of a multiple filament, in which conductive regions are shown in beige and the surrounding, insulating oxide is shown in blue. There appear to be at least three constituents to the overall growth, although the smallest of these is only a few nanometres high. The main filament is very straight, although it becomes slightly conical as it approaches the bottom electrode. Note that a single slice has been removed due to high levels of noise that saturated the scan area. (b) to (e) Selected cross-sections through the filament, showing the appearance and convergence of multiple filaments. 117 slices with a lateral resolution of 7.0 nm were used in this figure.

This journal is © The Royal Society of Chemistry 2015

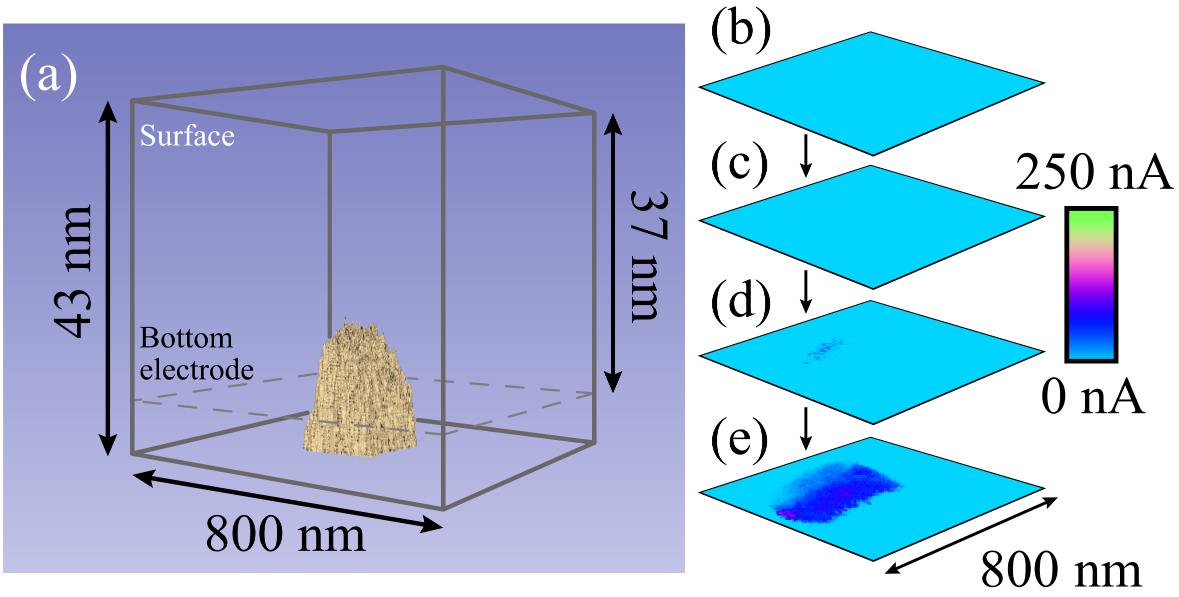

Fig. S7. Conductance tomography through pristine SiO_x. (a) Tomographic rendering in which conductive regions are shown in beige and the surrounding, insulating oxide is shown in blue. Near the bottom of the etch trench a conductive region appears, corresponding to the exposure of TiN by the CAFM tip. The height of the conductive region is relatively large, extending slightly into the insulating SiO_x. This is likely a result of a tunnelling current as the remaining SiO_x thickness between the tip and bottom electrode decreases. Additionally, we found the TiN to be slower to etch through than the SiO_x. Thus the height of the conductive region is likely to be overestimated. The conductive region does not extend over the entire measurement area. This may be a result of tip wear, the accumulation of insulating debris or an inhomogeneity in the TiN thickness. (b) to (e) Selected cross-sections through the SiO_x, showing the emergence of the bottom electrode towards the later slices of the scan. 206 slices with a lateral resolution of 3.1 nm were used in this figure.

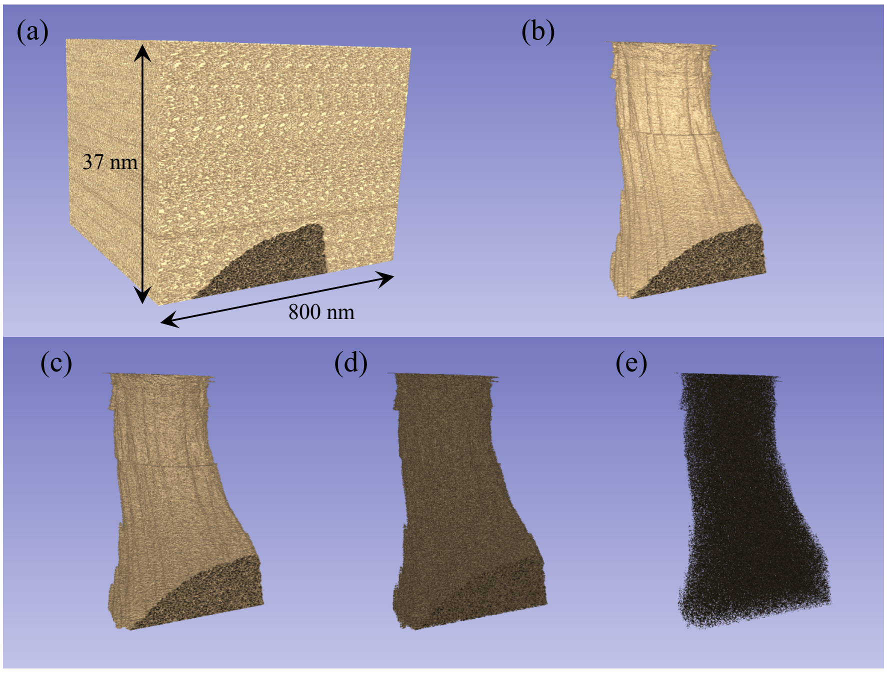

Fig. S8. Different threshold levels as applied to the filament of Figs. 3 and S3. (a) No thresholding applied. The filament is not visible in the measured region due to background noise. (b) By removing the noise floor of around 75 pA the filament is made visible. (c) Raising the thresholding to remove current up to around 50 nA reduces the clarity of the rendering as data at the edge of conductive regions is removed. (d) Thresholding at around 100 nA removes much of the definition of the filament as more internal regions are removed. (e) Thresholding to leave only values of current close to 200 nA results in a very unclear image with no definition. Here, only regions of maximum current are shown, corresponding to the core of the filament. In this case, the majority of the cross-sectional area of the filament corresponds to the core. Lower current values are confined to a small edge region.

 This journal is © The Royal Society of Chemistry 2015
